# Supplementary material for: The interactome of intact mitochondria by cross-linking mass spectrometry provides evidence for coexisting respiratory supercomplexes
Source: Mol Cell Proteomics. 2017 Dec 8;17(2):216–32. doi: 10.1074/mcp.RA117.000470 (PMC5795388; doi:10.1074/mcp.RA117.000470)
Supplement: Supplemental Data [file supp_RA117.000470_133922_0_supp_23978_fzffwf.zip › spectra_annotation/mito_spectra_annotation/303-1-3-1-16-1.pdf]

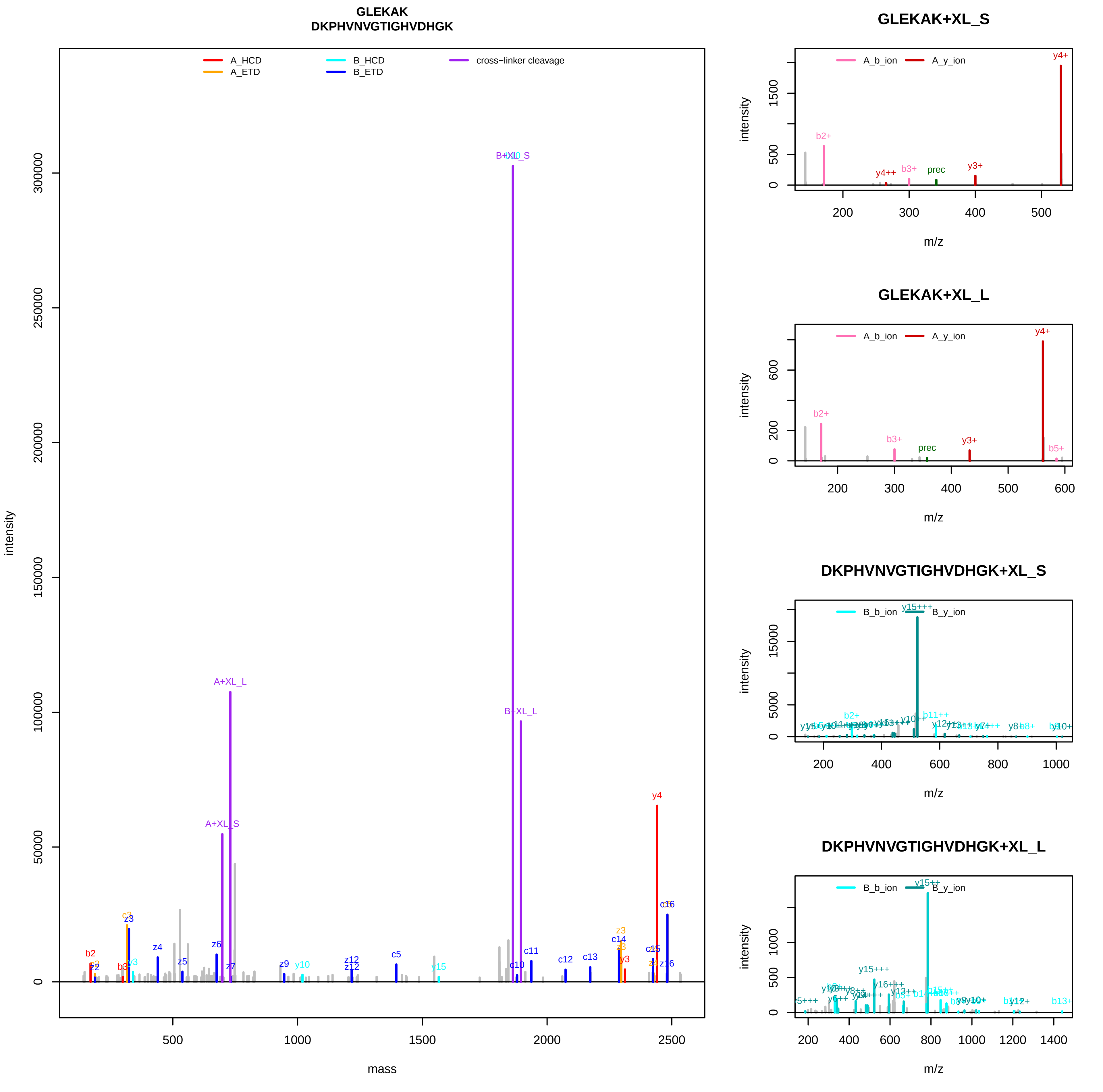
GLEKAK
DKPHVNVGTIGHVDHGK
A_HCD
A_ETD
B_HCD
B_ETD
cross−linker cleavage
B+XL_S
A+XL_L
B+XL_L
A+XL_S
y4
c16
z16
c15
c14
y3
c13
c12
c11
c10
c5
z12
y15
y10
z9
z7
z6
z5
z4
z3
c3
b2
z2
b3
intensity
mass
GLEKAK+XL_S
A_b_ion
A_y_ion
y4+
b2+
y4++
b3+
prec
y3+
m/z
GLEKAK+XL_L
b5+
DKPHVNVGTIGHVDHGK+XL_S
B_b_ion
B_y_ion
y15+++
b2+
b11++
y10++
y12++
y7+
y8+
b8+
y10+
DKPHVNVGTIGHVDHGK+XL_L
y15++
y15+++
y16+++
y13++
y6++
y8++
y9+
y10+
y12+
b13+
